# Phase II dose titration study of regorafenib in progressive unresectable metastatic colorectal cancer

**DOI:** 10.1038/s41598-022-24057-0

**Published:** 2023-02-09

**Authors:** Takeshi Kato, Toshihiro Kudo, Yoshinori Kagawa, Kohei Murata, Hirofumi Ota, Shingo Noura, Junichi Hasegawa, Hiroshi Tamagawa, Katsuya Ohta, Masakazu Ikenaga, Susumu Miyazaki, Takamichi Komori, Mamoru Uemura, Junichi Nishimura, Taishi Hata, Chu Matsuda, Taroh Satoh, Tsunekazu Mizushima, Yuko Ohno, Hirofumi Yamamoto, Yuichiro Doki, Hidetoshi Eguchi

**Affiliations:** 1 Department of Surgery, National Hospital Organization Osaka National Hospital, Osaka, Japan; 2 Department of Frontier Science for Cancer and Chemotherapy, Osaka University Graduate School of Medicine, Suita, Japan; 3 Department of Colorectal Surgery, Kansai Rosai Hospital, Amagasaki, Japan; 4 Department of Digestive Surgery, Ikeda City Hospital, Ikeda, Japan; 5 Department of Surgery, Osaka Rosai Hospital, Sakai, Japan; 6 Department of Surgery, Otemae Hospital, Osaka, Japan; 7 Department of Gastroenterological Surgery, Higashiosaka City Medical Center, Higashiosaka, Japan; 8 Department of Surgery, Osaka General Medical Center, Osaka, Japan; 9 Department of Gastroenterological Surgery, Graduate School of Medicine, Osaka University, Suita, Japan; 10 Department of Mathematical Health Science, Graduate School of Medicine, Osaka University, Suita, Japan

**Keywords:** Colorectal cancer, Chemotherapy, Targeted therapies

## Abstract

Regorafenib has shown significant survival benefit as a salvage therapy for colorectal cancer; however, its starting dose has been controversial in recent studies. Therefore, we conducted a prospective study on the efficacy and safety of the dose reduction of regorafenib to 120 mg. Patients received 120 mg regorafenib once per day for 3 weeks, followed by a 1-week off-treatment period. The primary endpoint was the investigator-assessed disease control rate (DCR). Sixty patients were registered, and the DCR was 38.3% with a median progression-free survival of 2.5 months (95% confidence interval [CI] 1.9–3.7) and median overall survival of 10.0 months (95% CI 6.9–15.2). Common grade 3–4 adverse events were hand-foot skin reaction and hypertension (20.0% each). The results of administration of 120 mg regorafenib as the starting dose are consistent with reports from prior phase III trials, which used starting doses of 160 mg. This lower initiating dose of regorafenib may be beneficial to certain patient populations. This clinical trial was registered in the UMIN Clinical Trials Registry (UMIN-CTR number UMIN000018968, registration date: 10/09/2015).

## Introduction

Regorafenib is an oral multitargeted kinase inhibitor that blocks the activity of several protein kinases associated with angiogenesis, oncogenesis, and tumour microenvironment^1^. The international multicentre Phase III CORRECT trial compared regorafenib with placebo in patients with metastatic colorectal cancer (mCRC) that had progressed following all available standard therapies or were unable to tolerate standard therapies^2^. Based on the results of the CORRECT trial, regorafenib is recommended by international guidelines as one of the standard drugs for treating mCRC^3–7^.

The standard starting dose of regorafenib is 160 mg/day regardless of patient height, body weight, race, and other parameters. However, continuous treatment at this dose tends to be complicated in routine medical care by its associated side effects, and therefore, the treatment dose often needs to be reduced to ≤ 120 mg/day. In the CORRECT trial, only 20% of participants required a reduction in regorafenib dose^2^. However, up to 49% of participants in the CONSIGN Phase IIIb study were reported to require a regorafenib dose reduction and 9% of them discontinued treatment because of drug-related adverse events (AEs), representing a total of 60% of participants who needed treatment modifications (dose reduction or interruption)^8^. Consequently, the CONSIGN study recognised the importance of treatment modification. Moreover, a systematic review of regorafenib for the treatment of mCRC reflected a similar realisation while also highlighting the need for a new treatment strategy to replace the existing regimen consisting of 160 mg/day for 3 weeks on followed by 1 week off treatment^9^. In the present study, we sought to determine whether the safety of continued regorafenib treatment could be enhanced, while maintaining a similar level of efficacy, by starting with a reduced dose.

## Materials and methods

### Study design and participants

This REGOCC-12 trial (UMIN registry identifier: UMIN000018968) is a multicentre, single-arm, open-label, Phase II exploratory study conducted at 31 institutions in Japan. Ethical approval of the study protocol was granted by Osaka University Research Ethics Review Committee. The trial was conducted in accordance with the principles of the Declaration of Helsinki and the Good Clinical Practice guidelines. All patients provided written informed consent before enrolment.

The key inclusion criteria were age ≥ 20 years, unresectable or recurrent colorectal adenocarcinoma, and disease progression during standard chemotherapy or within 3 months after the last administration of standard chemotherapy. Standard chemotherapy involved fluoropyrimidine, oxaliplatin, irinotecan, bevacizumab, and anti-epidermal growth factor receptor (anti-EGFR; only in patients with wild type [WT] RAS). Patients previously treated with trifluridine/tipiracil (TFTD) were not included. Eligible patients also had one or more measurable lesions based on the Response Evaluation Criteria in Solid Tumors (RECIST, version 1.1) guideline^10^, an Eastern Cooperative Oncology Group (ECOG) performance status (PS) of 0–1, and adequate organ function.

### Procedures

Regorafenib was orally administered at a dose of 120 mg per patient once daily after meals for 3 weeks (day 1–21), followed by a 1-week off-treatment period (day 22–28). This 4-week period was considered one cycle. Dose reduction or interruption was allowed during treatment based on the severity of the regorafenib-related AEs. Dose modification was initiated for AEs > grade 2, except for hand-foot skin reaction, hypertension, and increases in aspartate aminotransferase (AST), alanine aminotransferase (ALT), or total bilirubin (T-Bil) (Supplementary Table S1). For patients who required a dose interruption, regorafenib treatment was only resumed during the oral administration period of each cycle (day 1–21).

If AEs ≥ grade 2 were not observed in cycle 1, a dose increase to 160 mg/day in cycle 2 and beyond was allowed. However, when only AEs ≥ grade 1 for AST, ALT, or bilirubin increases were newly observed, the regorafenib dose was not increased. Treatment continued until tumour progression, unacceptable side-effects, or withdrawal of consent occurred. Antitumor response was evaluated by each investigator every 8 weeks according to the RECIST guidelines. AEs were assessed by investigators and reported according to the National Cancer Institute (NCI) Common Terminology Criteria for Adverse Events (CTCAE, version 4.0)^11^.

### Study endpoints

The primary endpoint was the investigator-assessed disease control rate (DCR) for the initial 120 mg/day dosage, defined as the proportion of patients with best overall complete response (CR), partial response (PR), or stable disease (SD) based on the RECIST guidelines. DCR was analysed using the intention-to-treat analysis.

Secondary endpoints were progression-free survival (PFS), overall survival (OS), objective response rate (ORR), duration of treatment, and safety profile. Safety analysis was performed in the safety population, which comprised patients who received ≥ one dose of regorafenib.

### Statistical considerations

With a 2-year patient accrual period, recruitment of approximately 60 participants was expected. According to the CORRECT study, the DCR of the regorafenib 160 mg/day-treated group was 41%^2^, while the corresponding values for the Japanese subset and placebo groups were 40% and 15%, respectively. Therefore, we set the threshold value at 27% considering that it would be clinically meaningless if the DCR deteriorated by 30% or more. Considering these results with an expected DCR of up to 40% with regorafenib 120 mg/day and a threshold DCR of 27%, a sample proportion test was possible using normal approximation, a one-sided significance level of 10%, and detection power of > 80%.

## Results

Sixty patients were enrolled and registered in this study between September 2015 and March 2017, and 58 of them began the study treatment as 2 patients were unable to start treatment due to rapid disease progression. Table 1 shows patient characteristics.

**Table 1:** Baseline characteristics.

	N = 60	
Median age (range), years	68.5 (47–80)	
Median height (range), cm	159 (146–172)	
Median body weight (range), kg	55.9 (39.6–76.1)	
**Sex, n**		
Male	30 (50%)	
Female	30 (50%)	
**ECOG performance status, n**		
0	42 (70%)	
1	18 (30%)	
**Primary location, n**		
Cecum	2 (3.3%)	Right, 18.3%
Ascending colon	9 (15%)	
Transverse colon	3 (5%)	
Descending colon	1 (1.7%)	Left, 76.6%
Sigmoid colon	27 (45%)	
Rectum	18 (30%)	
**Histology, n**		
Well-diff. adenoca	22 (36.7%)	
Moderately diff. adenoca	36 (60%)	
Mucinous adenoca	2 (3.3%)	
**RAS status, n**		
Wild-type	33 (55%)	
Mutant	27 (45%)	
**Metastatic sites, n**		
Liver	35 (67.3%)	
Lung	28 (53.8%)	
Peritoneum	15 (28.8%)	

Ratio of right and left colon, excluding transverse colon was calculated for site of primary lesion.

*ECOG* Eastern Cooperative Oncology Group, *diff.* differentiated; adenoca., adenocarcinoma.

The mean (standard deviation) and median (range) regorafenib treatment periods were 2.9 (2.2) and 2.0 (0.2–11.0) months, respectively, whereas the mean actual daily dose and dose intensity were 108.9 (16.4) mg and 71.0% (22.6%) when 120 mg was taken as 100%, respectively (Supplementary Table S2).

**Figure 1:**
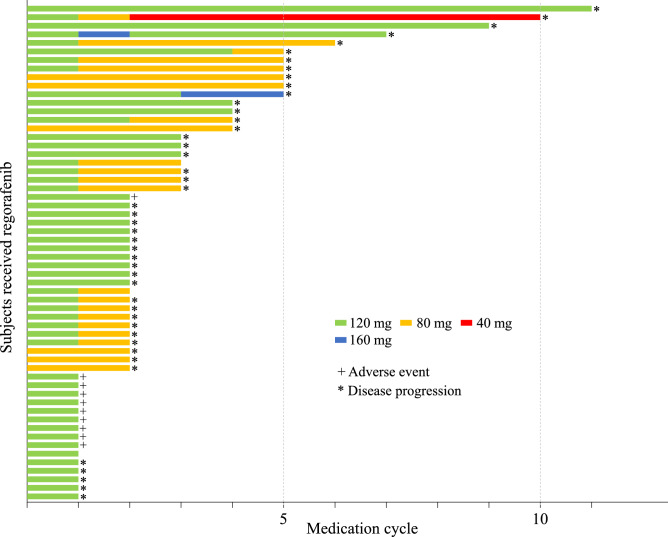
Swimmer plot of regorafenib dosing history. A summary of the weekly dosing history of each patient up to cycle 11 is shown. The symbol at the right end of each bar indicates the reason for treatment termination: ^+^due to adverse event, ^*^due to disease progression (N = 58).

Regarding regorafenib dose reduction, 6 patients during cycle 1, 15 during cycle 2, 1 during cycle 3, and 1 during cycle 5 had their doses reduced from 120 to 80 mg. Moreover, one patient required a dose reduction to 40 mg from cycle 3 to 10 with disease control. In contrast, it was possible to increase the dose to 160 mg in two patients. Specifically, one patient had the dose increased to 160 mg at cycle 2, which was then returned to 120 mg from cycle 3 because of a grade 2 hand-foot skin reaction, and the other had the dose increased to 160 mg at cycle 4, which was discontinued because progressive disease (PD) was observed at the end of cycle 5 (Fig. 1).

**Figure 2:**
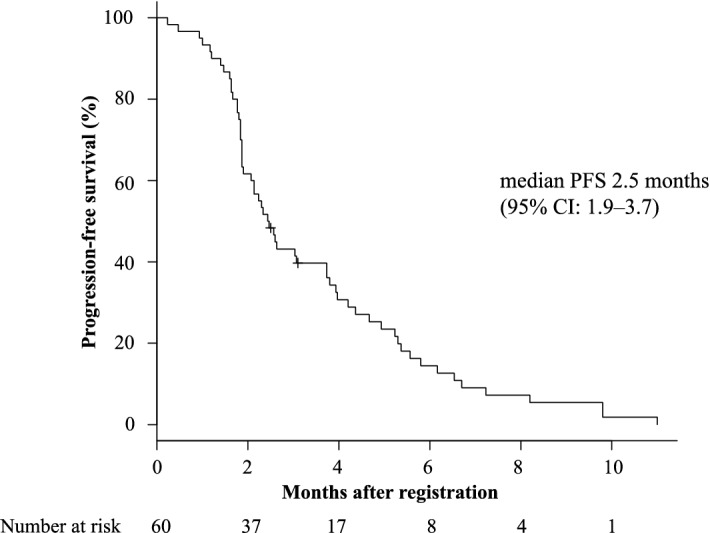
Progression free survival (PFS) during the study. *CI* confidence interval.

**Figure 3:**
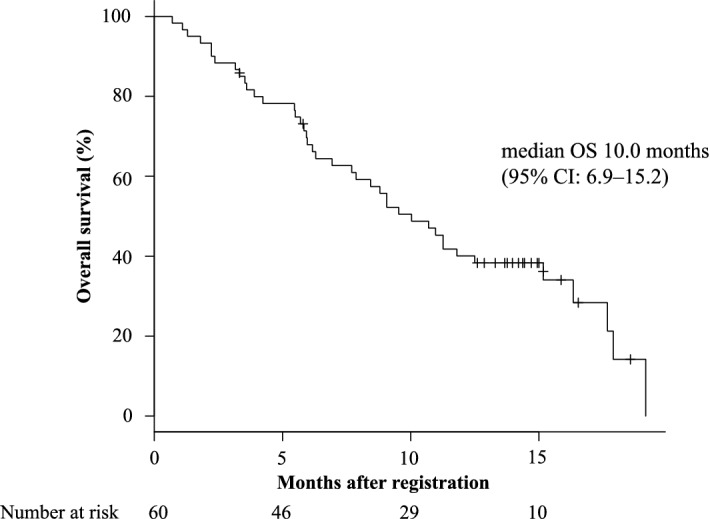
Overall survival (OS) during the study. *CI* confidence interval.

No patients showed CR or PR in this study, but SD and PD were observed in 23 (38.3%) and 36 (60%) patients, respectively, while one (1.7%) was not evaluable. As to the primary endpoint, DCR was 38.3% (90% confidence interval [CI] 28.0–48.7). The median PFS and OS were 2.5 (95% CI 1.9–3.7) months and 10.0 (95% CI 6.9–15.2) months (Figs. 2 and 3), respectively. The 12-month survival rate was 40.0% (95% CI 27.5–52.3). The median survival according to RAS oncogene status did not show any difference between treatments with 11.3 (95% CI 6.9–17.9) months and 10.7 (95% CI 5.8–15.10) months (p = 0.504) for the WT and mutant RAS, respectively (Supplementary Fig. S1). In contrast, the median survival according to primary lesion site showed a significant difference with 11.3 (95% CI 8.4–17.7) months and 6.3 (95% CI 2.2–10.7) months (p = 0.00807) for the left and right side of the colon, respectively (Supplementary Fig. S2).

In this study, 97% of patients showed some form of AEs, which are listed in Table 2. The most frequent AE was hand-foot skin reaction, and the most frequent events of all grades observed in over 50% of patients, other than hand-foot skin reaction (70%), were hypertension (57%) and increased AST (60%). The AEs ≥ grade 3 observed in ≥ 5% of patients were hand-foot skin reaction (20%), hypertension (20%), increased AST and ALT (10% each), hyperbilirubinemia (7%), and proteinuria (5%). Although 41 patients died during the study period, all mortalities were due to exacerbation of the primary disease.

**Table 2:** Treatment-emergent adverse events.

	Any grade, n (%)	≥ Grade 3, n (%)
Any TEAE	58 (97)	33 (55)
**Clinical adverse event**		
Fatigue	25 (42)	0 (0)
Hand-foot skin reaction	42 (70)	12 (20)
Diarrhoea	10 (17)	0 (0)
Anorexia	9 (15)	0 (0)
Voice changes	3 (5)	0 (0)
Hypertension	34 (57)	12 (20)
Oral mucositis	8 (13)	1 (2)
Rash or desquamation	8 (13)	1 (2)
Nausea	1 (2)	0 (0)
Fever	5 (8)	0 (0)
Taste alteration	2 (3)	0 (0)
Vomiting	0 (0)	0 (0)
Headache	1 (2)	0 (0)
Pain, abdominal	2 (3)	1 (2)
**Laboratory abnormalities**		
Thrombocytopenia	25 (42)	1 (2)
Hyperbilirubinemia	21 (35)	4 (7)
Proteinuria	6 (10)	3 (5)
Anaemia	27 (45)	2 (3)
AST elevation	36 (60)	6 (10)
ALT elevation	25 (42)	6 (10)

Safety population; N = 58.

*AST* aspartate aminotransferase, *ALT* alanine aminotransferase, *TEAE* treatment-emergent adverse event.

In this study, 41.4, 72.4, and 75.9% of patients required a regorafenib dose reduction, interruption, and modification, respectively, because of treatment-related AEs (TRAEs). Moreover, 10 of the 58 patients permanently discontinued treatment because of AEs, including 9 patients (15.5%) who had TRAEs (Supplementary Table S3). In addition, eight of these nine patients experienced TRAEs during cycle 1 and one patient during cycle 2. In most cases, TRAEs leading to permanent discontinuation appeared as a combination of multiple ≥ grade 2 factors (e.g. grade 3 hand-foot skin reaction plus grade 2 thrombocytopenia). In contrast, two of the nine patients decided to discontinue treatment because of liver damage alone. Of the 58 patients, three withdrew their consent, including two who did so because of hand-foot skin reaction. The dose was reduced from 120 to 80 mg for 24 patients, with the most common reason being hand-foot skin reaction, which was complained by nine patients (Supplementary Table S4).

## Discussion

This study showed that even when regorafenib treatment was initiated with a reduced dose of 120 mg, it was still possible to achieve a DCR similar to that obtained using a starting dose of 160 mg. The lower limit of the 90% CI of the DCR exceeded the 27% value that was previously set, indicating that the primary endpoint was achieved in this study. The median PFS was approximately the same duration or even longer than that of the CORRECT study^2^, and the median survival period reached 10 months, indicating that the results might be adequate to support the tested regimen being a salvage treatment, even as a single-arm Phase II study.

The DCR threshold was set to 27%, based on the actual clinical patient group (various clinical parameters, such as PS, tend to be worse overall than in the patient group that can participate in clinical trials). We assumed that regorafenib would be clinically significant if the DCR value did not decrease to below 70% of the DCR value (40%) in the Japanese subset^22^ of the CORRECT study. In fact, the CORRELATE study, an observational study in routine clinical practice, showed a DCR of 26%, even though the study included only 13% Asians^12^. Although the prospective CONCUR trial in Asian people reported a DCR of 51%, this study did not include Japanese patients^23^.

In addition, although the treatment starting dose was reduced to 120 mg, the dose intensity was at the same level as that of the CORRECT study. This observation suggests that because the starting doses differed between the two studies, the actual daily dose in the present study was lower than that in the CORRECT study. Prior to initiating our study, we predicted that setting a low starting dose would reduce the incidence rate of serious AEs (caused by a high dose, such as 160 mg). In addition, we predicted that the dose intensity in this study would be higher than that in the CORRECT study (78.9%) because the final treatment dose would achieve the same level of efficacy regardless of whether the starting dose was 160 mg or 120 mg.

However, this was not our finding, as the dose intensity in the present study was 71.0%. In the CORRECT study, 75.6% of patients needed a dose modification, and the present study showed similar but not lower values, with 75.9% of patients needing dose modifications. The incidence rate of AEs ≥ grade 3 in the present study, which was 55%, may seem comparable to that reported in the CORRECT study (51%) at first glance; however, it is necessary to note that while the present study did not collect TRAE data but treatment-emergent AEs (TEAEs) data, the CORRECT study findings were based on TRAEs.

The CORRECT and CONSIGN^8^ studies, with a starting treatment dose of 160 mg, reported TEAEs/TRAEs ≥ grade 3 of 78%/51% and 66%/57%, respectively. In contrast, the CORRELATE study^12^, which permitted dose reductions from the beginning, showed TEAEs and TRAEs ≥ grade 3 in 45% and 35% of patients, respectively. The values of the CORRELATE study were lower than those of the CORRECT and CONSIGN study. From these findings, the TRAE value in our study may be lower than 55%.

It is also important to note that while a certain number of ≥ grade 3 fatigue and diarrhoea events occurred in the CORRECT and CONSIGN studies, none were observed in the present study. In salvage treatments where the quality of life (QOL) is considered important, having only a few AEs ≥ grade 3 with subjective symptoms may be more significant than prolongation of OS. Moreover, supportive care has become important in recent years suggesting the effectiveness of using corticosteroids for fatigue^13^. In contrast, the incidence rate of hypertension and hand-foot skin reaction was higher in the present study than it was in the CORRECT and CONSIGN studies. This suggests that initiating treatment with a reduced dose may not necessarily decrease the incidence of certain side effects. In fact, according to post-marketing surveillance (PMS) in Japan, it is reported that the frequency of AEs was the same as that of the 160 mg dose depending on the type of AEs even after reduced starting dose^14^.

Our study did not show a clear reduction in the frequency of AEs by lowering the starting dose to 120 mg. However, recently, the importance of adjusting the dose of regorafenib and surviving the first two cycles immediately after the start of treatment, during which time the frequency of incidence of side effects is high, has been emphasised^8,9,15^. In fact, most cases of treatment discontinuation due to TRAEs in this study occurred during cycle 1, and the general consensus is that the strategy for managing AEs during the early stages of cycle 1 to cycle 2 is important. The most common reason for dose reduction in this study was hand-foot skin reaction. Considering that two patients withdrew their consent because of hand-foot skin reaction, effectively controlling this AE may decide the success or failure of regorafenib treatments. Moreover, the ReDOS study, whose primary endpoint was the proportion of patients who completed two cycles of treatment and initiated the third cycle, reported that dose titration of regorafenib may achieve longer survival than starting the treatment with 160 mg^16^. These facts also suggest that it is important not to cause discontinuation due to AEs by starting from a reduced dose.

Interestingly, one patient exhibited long-term disease control even after the regorafenib dose was reduced to 40 mg. Unfortunately, there are no data on whether doses below 80 mg/day provide a tumour shrinkage effect^17^. However, this suggests that the optimum dose varies between individual patients and 160 mg may not be a suitable starting dose for all patients. One study that used the same starting dose of 120 mg reported that the blood concentrations of regorafenib and its metabolites were low in patients who could continue treatments after the regorafenib dose was increased from 120 to 160 mg^18^. Moreover, it has been speculated that large individual differences exist in the capacity to metabolise regorafenib.

The OS in this study was 10 months, which is long for a salvage therapy. Moreover, 12-month survival was observed in 40.0% of patients, which was higher than the 24.3% in the CORRECT study. Of all 60 patients, 23 either could not or did not want to change to the subsequent treatment, but 36 did undergo some subsequent treatment. Of those administered subsequent treatments, 29 patients had used TFTD at some point, and 10 patients showed post-progression survival for 12 months or more; all of these patients had a history of TFTD use. The period of this study differed from that of the CORRECT study, and the increase in TFTD use was thought to have extended the survival post-progression and ultimately prolonged the OS in our study. Using fluoropyrimidine, oxaliplatin, and irinotecan is known to prolong survival^19^, but currently, it has been recognised that using up both regorafenib and TFTD in the salvage line is important for prolonging OS^20,21^.

Currently, no conclusion has been reached on whether the optimum starting dose of regorafenib should be 160 mg for all patients, and several outstanding issues need to be resolved, including relevant biomarkers, for this drug to be used most effectively. However, we hope that the results of this study will facilitate the establishment of the most effective administration strategy for regorafenib.

## Supplementary Information

Supplementary Information 1.

Supplementary Information 2.

Supplementary Information 3.

Supplementary Information 4.

Supplementary Information 5.

Supplementary Information 6.

## Data Availability

The data supporting the findings of this study are available on request from the corresponding author after approval from the Osaka University Research Ethics Review Committee. The data are not publicly available since they contain information that could compromise the patients’ privacy.
