# Supplementary material for: Phase II dose titration study of regorafenib in progressive unresectable metastatic colorectal cancer
Source: Sci Rep. 2023 Feb 9;13:2331. doi: 10.1038/s41598-022-24057-0 (PMC9911606; doi:10.1038/s41598-022-24057-0)
Supplement: Supplementary file 1 — Supplementary Information 1. [file 41598_2022_24057_MOESM1_ESM.pptx]

## Slide 1
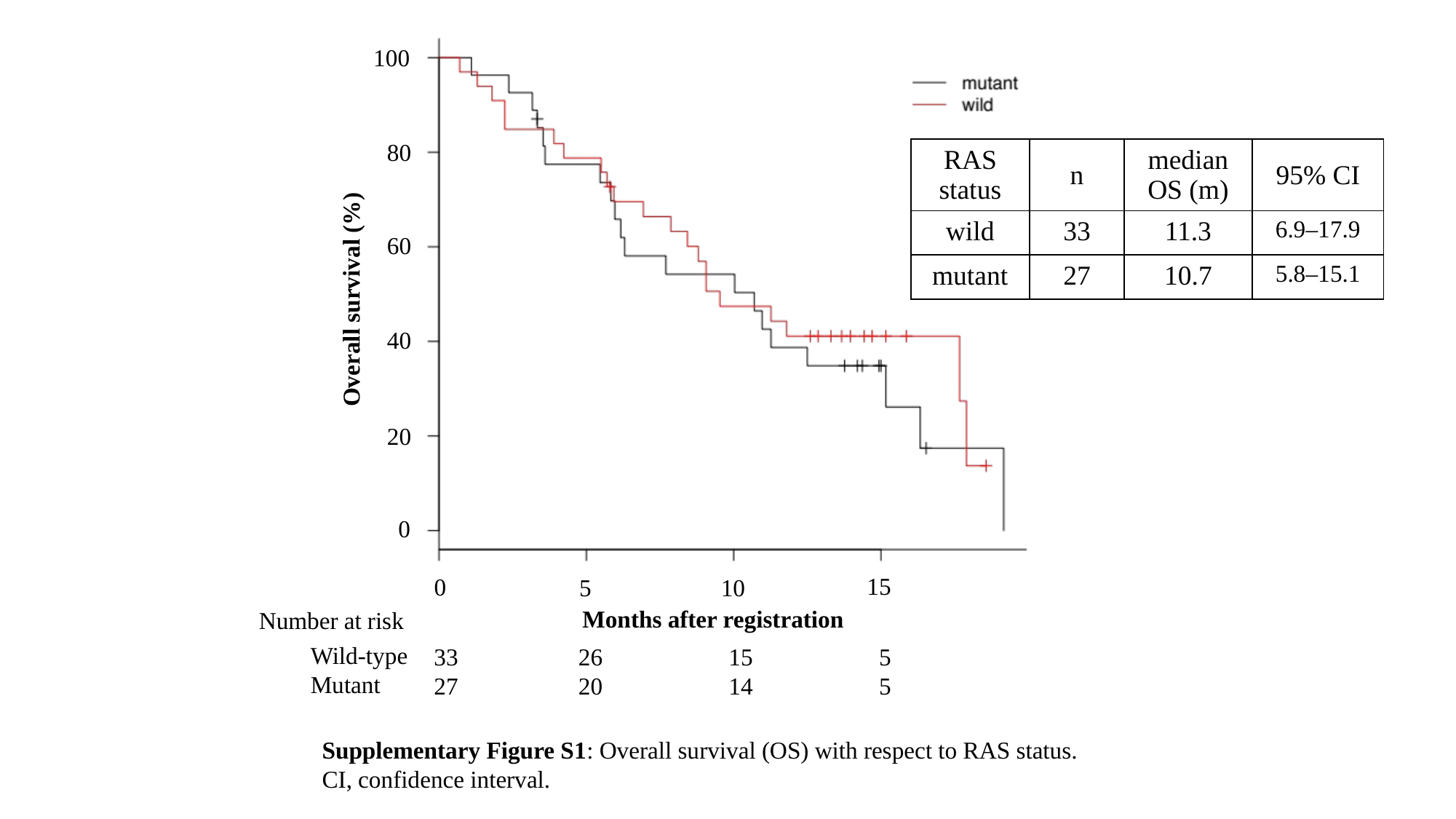

100
80
| RAS status | n | median OS (m) | 95% CI |
| --- | --- | --- | --- |
| wild | 33 | 11.3 | 6.9–17.9 |
| mutant | 27 | 10.7 | 5.8–15.1 |
Overall survival (%)
60
40
20
0
15
0
5
10
Months after registration
Number at risk
Wild-type
Mutant
33 26 15 5
27 20 14 5
Supplementary Figure S1: Overall survival (OS) with respect to RAS status.
CI, confidence interval.

## Slide 2
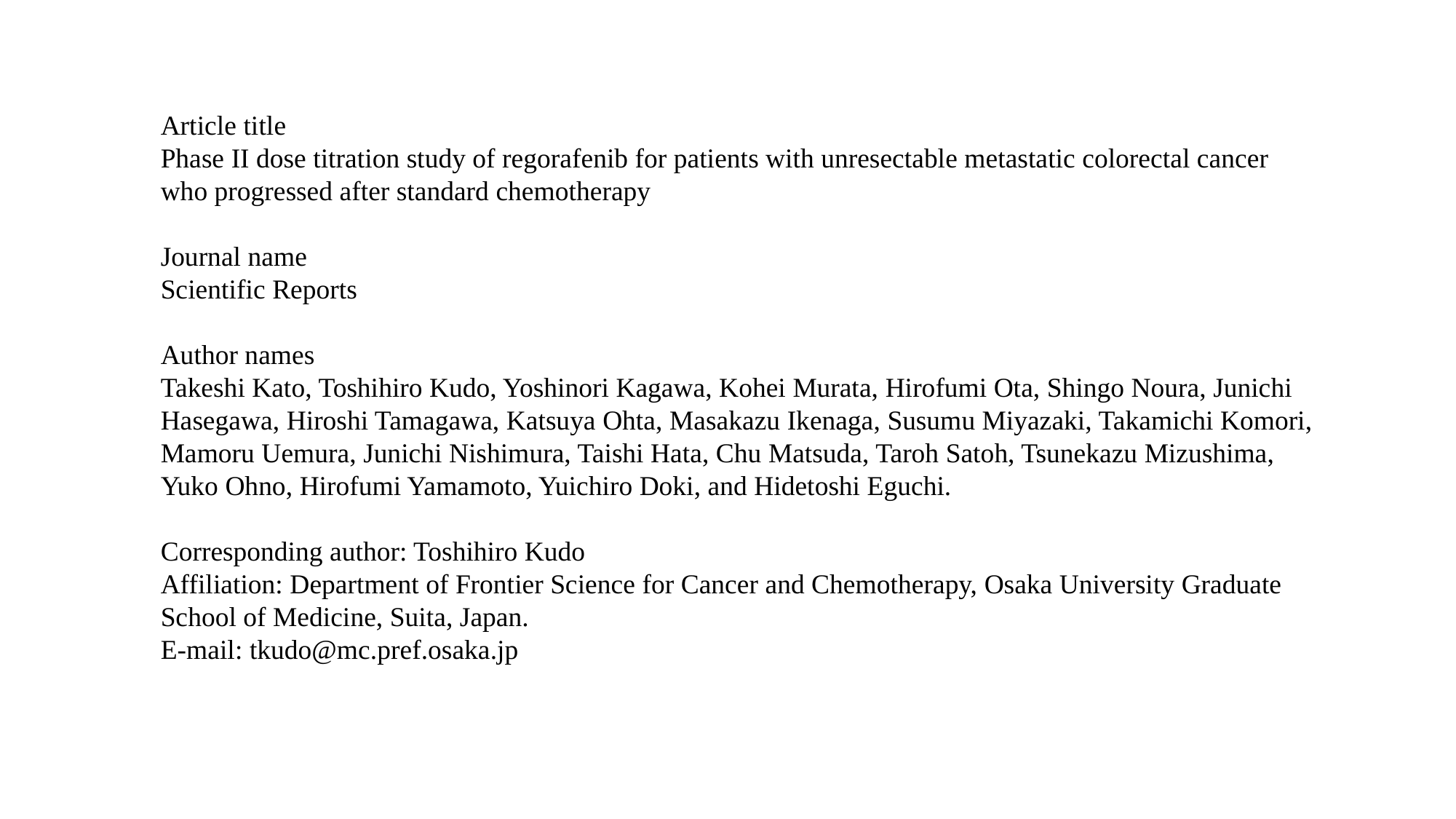

Article title
Phase II dose titration study of regorafenib for patients with unresectable metastatic colorectal cancer who progressed after standard chemotherapy
Journal name
Scientific Reports
Author names
Takeshi Kato, Toshihiro Kudo, Yoshinori Kagawa, Kohei Murata, Hirofumi Ota, Shingo Noura, Junichi Hasegawa, Hiroshi Tamagawa, Katsuya Ohta, Masakazu Ikenaga, Susumu Miyazaki, Takamichi Komori, Mamoru Uemura, Junichi Nishimura, Taishi Hata, Chu Matsuda, Taroh Satoh, Tsunekazu Mizushima, Yuko Ohno, Hirofumi Yamamoto, Yuichiro Doki, and Hidetoshi Eguchi.
Corresponding author: Toshihiro Kudo
Affiliation: Department of Frontier Science for Cancer and Chemotherapy, Osaka University Graduate School of Medicine, Suita, Japan.
E-mail: tkudo@mc.pref.osaka.jp
