# Supplementary material for: Phase II dose titration study of regorafenib in progressive unresectable metastatic colorectal cancer
Source: Sci Rep. 2023 Feb 9;13:2331. doi: 10.1038/s41598-022-24057-0 (PMC9911606; doi:10.1038/s41598-022-24057-0)
Supplement: Supplementary file 3 — Supplementary Information 3. [file 41598_2022_24057_MOESM3_ESM.docx]

| Variable | Descriptions | |
| --- | --- | --- |
| AST/ALT increase | | |
| AST or ALT ≤ 5 × ULN | Continue with same dose | |
| AST or ALT > 5 × ULN but < 20 × ULN | First time | Interrupt until resolved to < 3 × ULN, then reduce dose 1 level |
|  | Second time | Discontinue |
| AST or ALT ≥ 20 × ULN | Discontinue | |
| AST or ALT > 3 × ULN and T-Bil > 2 × ULN | Discontinue | |
| Hand-foot skin reaction | | |
| Grade 1 | Continue with same dose  with immediate supportive therapy | |
| Grade 2 | First time | Reduce dose 1 level and continue |
|  | Second and third times | Interrupt treatment until resolved to grade 0-1 and reduce dose 1 level |
|  | Fourth time | Discontinue |
| Grade 3 | First and second times | Interrupt until resolved to grade 0-1, then reduce dose 1 level |
|  | Third time | Discontinue |
| Grade 4 | Discontinue | |
| Hypertension | | |
| Grade 2 | Asymptomatic | Continue with same dose and start antihypertensive drugs |
|  | symptomatic | Interrupt until symptom resolution and blood pressure controlled |
| Grade 3 | Interrupt until symptom resolution and blood pressure controlled, then reduce dose 1 level | |
| Grade 4 | Discontinue | |

**Supplementary Table S1. Dose reduction and interruption criteria**

Article title

Phase II dose titration study of regorafenib for patients with unresectable metastatic colorectal cancer who progressed after standard chemotherapy

Journal name

Scientific Reports

Author names

Takeshi Kato, Toshihiro Kudo, Yoshinori Kagawa, Kohei Murata, Hirofumi Ota, Shingo Noura, Junichi Hasegawa, Hiroshi Tamagawa, Katsuya Ohta, Masakazu Ikenaga, Susumu Miyazaki, Takamichi Komori, Mamoru Uemura, Junichi Nishimura, Taishi Hata, Chu Matsuda, Taroh Satoh, Tsunekazu Mizushima, Yuko Ohno, Hirofumi Yamamoto, Yuichiro Doki, and Hidetoshi Eguchi.

Corresponding author: Toshihiro Kudo

Affiliation: Department of Frontier Science for Cancer and Chemotherapy, Osaka University Graduate School of Medicine, Suita, Japan.

E-mail: tkudo@mc.pref.osaka.jp
