# Supplementary material for: Phase II dose titration study of regorafenib in progressive unresectable metastatic colorectal cancer
Source: Sci Rep. 2023 Feb 9;13:2331. doi: 10.1038/s41598-022-24057-0 (PMC9911606; doi:10.1038/s41598-022-24057-0)
Supplement: Supplementary file 4 — Supplementary Information 4. [file 41598_2022_24057_MOESM4_ESM.docx]

|  | N = 58 |
| --- | --- |
| Overall duration of treatment (months) |  |
| Mean ± SD | 2.9 ± 2.2 |
| Median (range) | 2.0 (0.2–11.0) |
| Actual daily dose (mg) |  |
| Mean ± SD | 108.9 ± 16.4 |
| Median (range) | 120 (50.0–120) |
| Dose intensity (%) |  |
| Mean ± SD | 71.0 ± 22.6 |
| Median (range) | 68.3 (28.6–100) |
| Drug-related AE leading to: |  |
| Dose modification (%)^*^ | 75.9 |
| Dose reduction (%) | 41.4 |
| Dose interruption (%) | 72.4 |
| Permanent discontinuation (%) | 15.5 |

**Supplementary Table S2. Drug Exposure and Disposition**

*Dose modification includes reduction and interruption.

Abbreviations: SD, standard deviation; AE, adverse event.

Article title

Phase II dose titration study of regorafenib for patients with unresectable metastatic colorectal cancer who progressed after standard chemotherapy

Journal name

Scientific Reports

Author names

Takeshi Kato, Toshihiro Kudo, Yoshinori Kagawa, Kohei Murata, Hirofumi Ota, Shingo Noura, Junichi Hasegawa, Hiroshi Tamagawa, Katsuya Ohta, Masakazu Ikenaga, Susumu Miyazaki, Takamichi Komori, Mamoru Uemura, Junichi Nishimura, Taishi Hata, Chu Matsuda, Taroh Satoh, Tsunekazu Mizushima, Yuko Ohno, Hirofumi Yamamoto, Yuichiro Doki, and Hidetoshi Eguchi.

Corresponding author: Toshihiro Kudo

Affiliation: Department of Frontier Science for Cancer and Chemotherapy, Osaka University Graduate School of Medicine, Suita, Japan.

E-mail: tkudo@mc.pref.osaka.jp
