# Supplementary material for: Phase II dose titration study of regorafenib in progressive unresectable metastatic colorectal cancer
Source: Sci Rep. 2023 Feb 9;13:2331. doi: 10.1038/s41598-022-24057-0 (PMC9911606; doi:10.1038/s41598-022-24057-0)
Supplement: Supplementary file 5 — Supplementary Information 5. [file 41598_2022_24057_MOESM5_ESM.docx]

| Number of patients | Treatment-related adverse events (TRAEs) leading to discontinuation |
| --- | --- |
| 1 | Grade 2 Hand-foot skin reaction  Grade 2 Oral mucositis  Grade 1 Anaemia |
| 2 | Grade 3 Blood bilirubin increased  Grade 3 Oral mucositis  Grade 3 Rash (Erythema multiforme) |
| 3 | Grade 2 Blood bilirubin increased  Grade 2 Aspartate aminotransferase increased  Grade 3 Encephalopathy |
| 4 | Grade 3 Blood bilirubin increased  Grade 3 Aspartate aminotransferase increased  Grade 3 Alanine aminotransferase increased  Grade 3 Hand-foot skin reaction  Grade 2 Hypertension |
| 5 | Grade 2 Fever  Grade 1 Aspartate aminotransferase increased  Grade 1 Alanine aminotransferase increased  Grade 1 Diarrhoea |
| 6 | Grade 3 Proteinuria  Grade 3 Hand-foot skin reaction |
| 7 | Grade 2 Blood bilirubin increased  Grade 4 Aspartate aminotransferase increased  Grade 3 Alanine aminotransferase increased |
| 8 | Grade 3 Hypertension  Grade 3 Hand-foot skin reaction  Grade 2 Thrombocytopenia |
| 9* | Grade 4 Aspartate aminotransferase increased  Grade 4 Alanine aminotransferase increased |

**Supplementary Table S3. Reason for Discontinuation**

*Occurred during cycle 2.

Article title

Phase II dose titration study of regorafenib for patients with unresectable metastatic colorectal cancer who progressed after standard chemotherapy

Journal name

Scientific Reports

Author names

Takeshi Kato, Toshihiro Kudo, Yoshinori Kagawa, Kohei Murata, Hirofumi Ota, Shingo Noura, Junichi Hasegawa, Hiroshi Tamagawa, Katsuya Ohta, Masakazu Ikenaga, Susumu Miyazaki, Takamichi Komori, Mamoru Uemura, Junichi Nishimura, Taishi Hata, Chu Matsuda, Taroh Satoh, Tsunekazu Mizushima, Yuko Ohno, Hirofumi Yamamoto, Yuichiro Doki, and Hidetoshi Eguchi.

Corresponding author: Toshihiro Kudo

Affiliation: Department of Frontier Science for Cancer and Chemotherapy, Osaka University Graduate School of Medicine, Suita, Japan.

E-mail: tkudo@mc.pref.osaka.jp
