# Supplementary material for: Phase II dose titration study of regorafenib in progressive unresectable metastatic colorectal cancer
Source: Sci Rep. 2023 Feb 9;13:2331. doi: 10.1038/s41598-022-24057-0 (PMC9911606; doi:10.1038/s41598-022-24057-0)
Supplement: Supplementary file 6 — Supplementary Information 6. [file 41598_2022_24057_MOESM6_ESM.docx]

|  | Patient number and grade of adverse event (AE)  leading to dose modification | |
| --- | --- | --- |
|  | Single cause | Multiple cause |
| Hand-foot skin reaction | G3, G3, G3, G3, G3, G2, G2, G2, G2 | G2^†1^, G3^†3^ |
| Aspartate aminotransferase increased  or  Alanine aminotransferase increased | G3 | G2^†2^, G1^†4^ |
| Blood bilirubin increased | G3 | G2^†2^, G1^†4^ |
| Hypertension | G3 | G3^†1^, G2^†5^ |
| Proteinuria | G3 | G2^†4^ |
| Rash or desquamation | G2 | G2^†5^ |
| Fatigue | G1 | G1^†6^ |
| Abdominal pain | G3 | none |
| Diarrhoea | G2 | G2^†3^ |
| Thrombocytopenia | none | G2^†5^ |
| Fever | G1 (sustained) | none |

**Supplementary Table S4. Reason for Dose Modification**

†Number in superscript represents the same patient.

Article title

Phase II dose titration study of regorafenib for patients with unresectable metastatic colorectal cancer who progressed after standard chemotherapy

Journal name

Scientific Reports

Author names

Takeshi Kato, Toshihiro Kudo, Yoshinori Kagawa, Kohei Murata, Hirofumi Ota, Shingo Noura, Junichi Hasegawa, Hiroshi Tamagawa, Katsuya Ohta, Masakazu Ikenaga, Susumu Miyazaki, Takamichi Komori, Mamoru Uemura, Junichi Nishimura, Taishi Hata, Chu Matsuda, Taroh Satoh, Tsunekazu Mizushima, Yuko Ohno, Hirofumi Yamamoto, Yuichiro Doki, and Hidetoshi Eguchi.

Corresponding author: Toshihiro Kudo

Affiliation: Department of Frontier Science for Cancer and Chemotherapy, Osaka University Graduate School of Medicine, Suita, Japan.

E-mail: tkudo@mc.pref.osaka.jp
